# Layer number dependent ferroelasticity in 2D Ruddlesden–Popper organic-inorganic hybrid perovskites

**DOI:** 10.1038/s41467-021-21493-w

**Published:** 2021-02-26

**Authors:** Xun Xiao, Jian Zhou, Kepeng Song, Jingjing Zhao, Yu Zhou, Peter Neil Rudd, Yu Han, Ju Li, Jinsong Huang

**Affiliations:** 1 Department of Applied Physical Sciences, University of North Carolina, Chapel Hill, NC 27599 USA; 2 Department of Nuclear Science and Engineering and Department of Materials Science and Engineering, Massachusetts Institute of Technology, Cambridge, MA 02139 USA; 3 Advanced Membranes and Porous Materials (AMPM) Center, Physical Sciences and Engineering Division, King Abdullah University of Science and Technology (KAUST), Thuwal, Saudi Arabia

**Keywords:** Materials for energy and catalysis, Nanoscience and technology

## Abstract

Ferroelasticity represents material domains possessing spontaneous strain that can be switched by external stress. Three-dimensional perovskites like methylammonium lead iodide are determined to be ferroelastic. Layered perovskites have been applied in optoelectronic devices with outstanding performance. However, the understanding of lattice strain and ferroelasticity in layered perovskites is still lacking. Here, using the in-situ observation of switching domains in layered perovskite single crystals under external strain, we discover the evidence of ferroelasticity in layered perovskites with layer number more than one, while the perovskites with single octahedra layer do not show ferroelasticity. Density functional theory calculation shows that ferroelasticity in layered perovskites originates from the distortion of inorganic octahedra resulting from the rotation of aspherical methylammonium cations. The absence of methylammonium cations in single layer perovskite accounts for the lack of ferroelasticity. These ferroelastic domains do not induce non-radiative recombination or reduce the photoluminescence quantum yield.

## Abstract (web summary)

Ruddlesden popper layered perovskites can be used in optoelectronic devices, but the understanding of their lattice strain as well as ferroelasticity is still lacking. Here, the authors find ferroelasticity in layered perovskites with layer number more than one and reveal its mechanism.

## Introduction

Ferroelasticity is the mechanical analog of ferroelectricity and ferromagnetism, describing a material possessing spontaneous strain within domain structures and the consequent strain-stress hysteretic behavior^1,2^. Ferroelastic domains with different orientations can coexist through connections by twin boundaries (TBs). These domains can then be switched by the application of external stress, which is analogous to ferroelectricity and the spontaneous switching of polarization in response to an external electric field^3–5^. The switching behavior triggered by external strain or stress enables dynamic tuning of material properties by inelastic strain engineering, particularly for applications in flexible electronics^6–8^. The study of ferroelasticity in organic-inorganic halide perovskites began with the observation of domain-like structures by various characterization techniques^9–13^. Using piezoresponse force microscopy (PFM), Hermes et al. reported the first observation of periodic domains in methylammonium lead iodide (MAPI) in 2016^9^. In the same year, we observed the striped contrast on the MAPI grains with photothermal induced resonance (PTIR)^13^. Following these observations, significant effort has been devoted to elucidating the origin of these domains^11,12,14^. While the ferroelectric nature of these domains is still hotly debated^11,12,14,15^, Strelcov et al. confirmed the domains to be ferroelastic through the in-situ observation of switching domains under a polarized optical microscope with external stress in MAPI thin films and single crystals^16^. Rothmann et al. then proceeded to study the twin structures in MAPI with low dose transmission electron microscopy (TEM) and selected area electron diffraction (SAED), identifying the twin boundaries to be {112} planes in the tetragonal phase^10^. Recently, we investigated the impact of these twin boundaries (TBs) on carrier transport and recombination properties of metal halide perovskites^17^. Using scanning photocurrent mapping and photoluminescence (PL) imaging, TBs were found to exhibit benign electronic behavior, i.e., neither blocking carrier transport across them nor introducing non-radiative recombination pathways. This benign nature indicates that ferroelasticity in MAPI can help relieve device stress^18^ in processing and service, making them more flexible without sacrificing carrier transport or device performance.

In addition to conventional three dimensional (3D) perovskite structures, like MAPI, layered or two-dimensional (2D) perovskites are emerging as stellar materials with significant structural differences and thus chemical and physical properties, which have been applied successfully for sensitive photodetectors^19^ and X-ray detectors^20^, efficient light-emitting diodes^21–23^, and solar cells with enhanced stability^24–28^. Compared to 3D perovskites, layered perovskites consist of inorganic octahedra sheets separated by organic cation spacers, and a general structure could be expressed as R_2_A_*n*-1_B_*n*_X_3*n*+1_, where R is organic cation spacer with long side chain to separate the inorganic layers, A is the organic cation in octoheral structures, B is the metal ion and X is the halide^29,30^. The number of inorganic sheets sandwiched between organic spacer cations (*n*) is variable, providing an alternative pathway to tune the perovskite structure and hence optoelectronic properties, like bandgap and carrier diffusivity^30,31^. However, in contrast to the intensive study of ferroelasticity in 3D perovskites, there is barely any characterization of lattice strain and ferroelasticity in layered perovskites. Whether layered perovskites even exhibit ferroelastic behavior has yet to be determined. If yes, it remains an open question whether it would affect the electronic properties like carrier transport or recombination kinetics in layered perovskite.

Here, we report evidence for the existence of ferroelasticity in layered perovskites via in-situ observations of ferroelastic domain motion in layered organic-inorganic perovskite single crystals under applied stress. Tuning the number of inorganic sheets between organic cations allowed us to determine the layer-dependent nature of ferroelasticity in layered perovskites. We found that ferroelasticity exists in layered perovskites with layer number *n* ≥ 2, while absent for *n* = 1. Density functional theory (DFT) calculations revealed that the ferroelasticity in layered perovskites is caused by the distortion of inorganic framework due to the rotation of aspherical A-site molecules, like the methylammonium (MA) cations. We also determined that the ferroelastic twin structures in layered perovskites have a negligible impact on carrier recombination kinetics with confocal photoluminescence mapping.

## Results

### Ferroelasticity in BAI-N2 single crystals

**Fig. 1:**
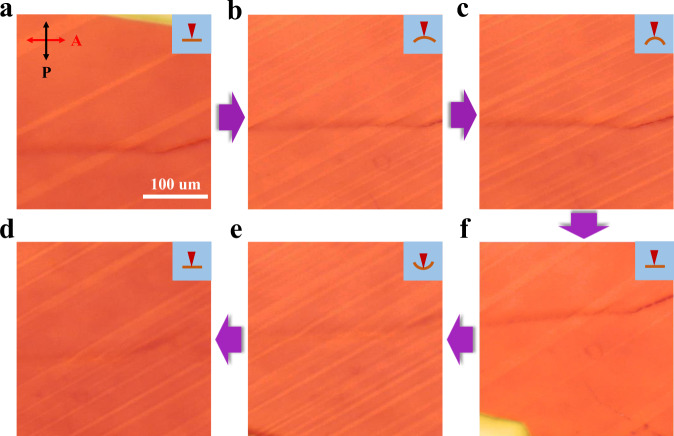
In-situ observation of ferroelastic domain motion in BAI-N2 single-crystalline layered perovskites by polarized optical microscope. **a**–**f** are in-situ polarized optical microscope images of the domain wall movement under applied stress in BAI-N2 crystals. The purple arrow indicates the bending sequence. The inset images show the bending states of the BAI-N2 single crystals and the red triangle represents the imaging direction. The red and black arrows in (**a**) indicate the analyzer and polarizer orientation, respectively. The dark line in the center is a surface scratch, which is chosen as a location marker.

The layered perovskite single crystals with high phase purity were synthesized by a recently developed space confinement method^28,32^. Solutions for BA_2_PbI_4_ (BAI-N1), BA_2_MAPb_2_I_7_ (BAI-N2), and BA_2_MA_2_Pb_3_I_10_ (BAI-N3) crystal growth were prepared by dissolving butylammonium iodide (BAI), methylammonium iodide (MAI), and lead iodide (PbI_2_), with respective molar ratios of 2:0:1, 2:1:2, and 2:2:3, in hydriodic acid (HI) at 130 °C. After inserting 150 μL precursor between two glass substrates (2 × 3 inches) at 130 °C, the growth temperature was gradually decreased to ~20 °C at a rate of 10 °C per h to produce thin layered perovskite crystals. The typical sizes for BAI-N1 single crystals are ~1 × 5 mm^2^ in area and ~10 μm in thickness, while the BAI-N2 and BAI-N3 single crystals are in similar size of ~4 × 4 mm^2^ in area and ~10 μm thick. The thin as-grown layered perovskite single crystals were analyzed with X-ray diffraction (XRD). Sharp diffraction peaks in the XRD pattern for all three samples were observed (Supplementary Fig. 1), which could be assigned to the same crystal plane family of (0 0 *k*) in the corresponding crystal structures, confirming the single crystalline, phase pure nature of the samples. The excellent crystallinity and layered structure of BAI-N2 could also be confirmed with cross-sectional TEM, as shown in Supplementary Fig. 2. The as-grown BAI-N2 crystals were transferred and fixed onto a flexible polydimethylsiloxane (PDMS) substrate, which allowed us to macroscopically apply stress and examine the ferroelasticity with the strain-driven motion of domain walls as shown in Supplementary Fig. 3. Ferroelastic domain patterns were captured using in situ polarized optical microscopy with a crossed Nicols configuration, an effective approach to distinguish differently oriented ferroelastic twin domains^16^. As shown in Fig. 1a, an obvious stripe pattern corresponding to ferroelastic twin domains was seen when the sample was flattened. Stress was then applied to the flexible substrate by bending it upward or downward with different curvatures for applying tensile or compressive strain to the BAI-N2 crystal. The bending states are indicated by the inset of Fig. 1a–f. A clear domain wall motion under different applied strain was observed (Fig. 1), confirming the ferroelastic nature of these domains. It should be noted that the three domain patterns of flattened states within the in-situ bending test (Fig. 1a, d, f) do not overlap, i.e., the domain patterns did not completely return to its original state when the applied stress was released. This lack of pattern memory agrees with standard ferroelastic systems^33^.

### Layer number dependent ferroelasticity

**Fig. 2:**
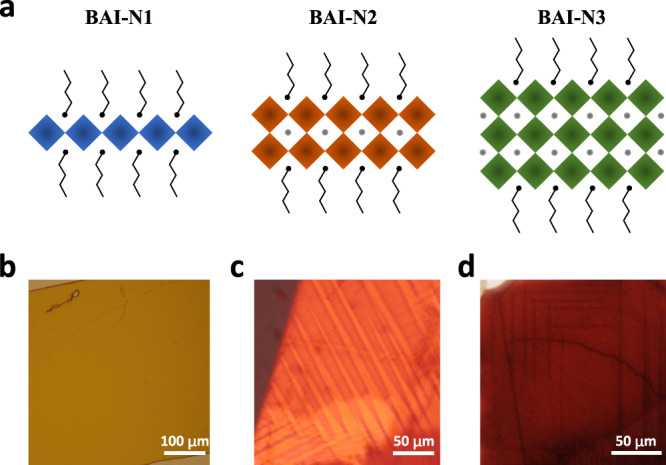
Layer number dependent ferroelasticity in layered perovskites. **a** Scheme of crystal structures for BAI-N1, BAI-N2, and BAI-N3; **b**–**d** corresponding polarized optical microscope images for BAI-N1, BAI-N2, and BAI-N3, respectively.

To investigate the relation between ferroelasticity and layered perovskites composition, we employed a polarized optical microscope on BAI-N1, BAI-N2, and BAI-N3, respectively, with tuning the number of inorganic layers inserted into organic spacer cations. The scheme of crystal structures for BAI-N1, BAI-N2, and BAI-N3 are presented in Fig. 2a. After transferred to flexible PDMS substrate and the bending test, the crystals are examined by the polarized optical microscope, and the optical images are shown in Fig. 2b–d. For BAI-N1 crystals, no obvious domain pattern was observed after applying stress. While for BAI-N2 and BAI-N3 samples, clear domain patterns could be resolved under the polarized optical microscope. To exclude morphology artifacts like texture, images under a non-polarized optical microscope were captured for comparison. As shown in Supplementary Figure 4, the domain patterns in BAI-N2 and BAI-N3 crystals could only be seen under the polarized rather than non-polarized optical microscope, which indicates that the domain patterns are caused by different crystallographic orientations instead of morphology. Even after applying a large stress before breaking them, the BAI-N1 samples showed no obvious pattern under a polarized or non-polarized optical microscope, suggesting that ferroelasticity is absent in BAI-N1.

The domain patterns under different temperature were captured to determine the Curie temperature. The sample’s temperature was controlled by a portable LINKAM thermal stage in the air. At each temperature, the sample was held for 6 min to stabilize the temperature before the polarized light optical image was taken. As shown in Supplementary Figure 5, clear domain patterns were observed in BAI-N2 crystal when the sample was heated until 150 °C, while some domains (indicated by the blue circle) disappeared when the temperature was above 180 °C. The twin domains showed up again when the sample was cooled down. It was found some domains (indicated by red rectangular) still existed even at 200 °C, which might be caused by defects which pinned twin boundaries or short holding time at this temperature (longer holding time induced sample degradation). Based on these observations, we infer that the Curie temperature should be around 180 °C.

### Origin for ferroelasticity in 2D perovskites

**Fig. 3:**
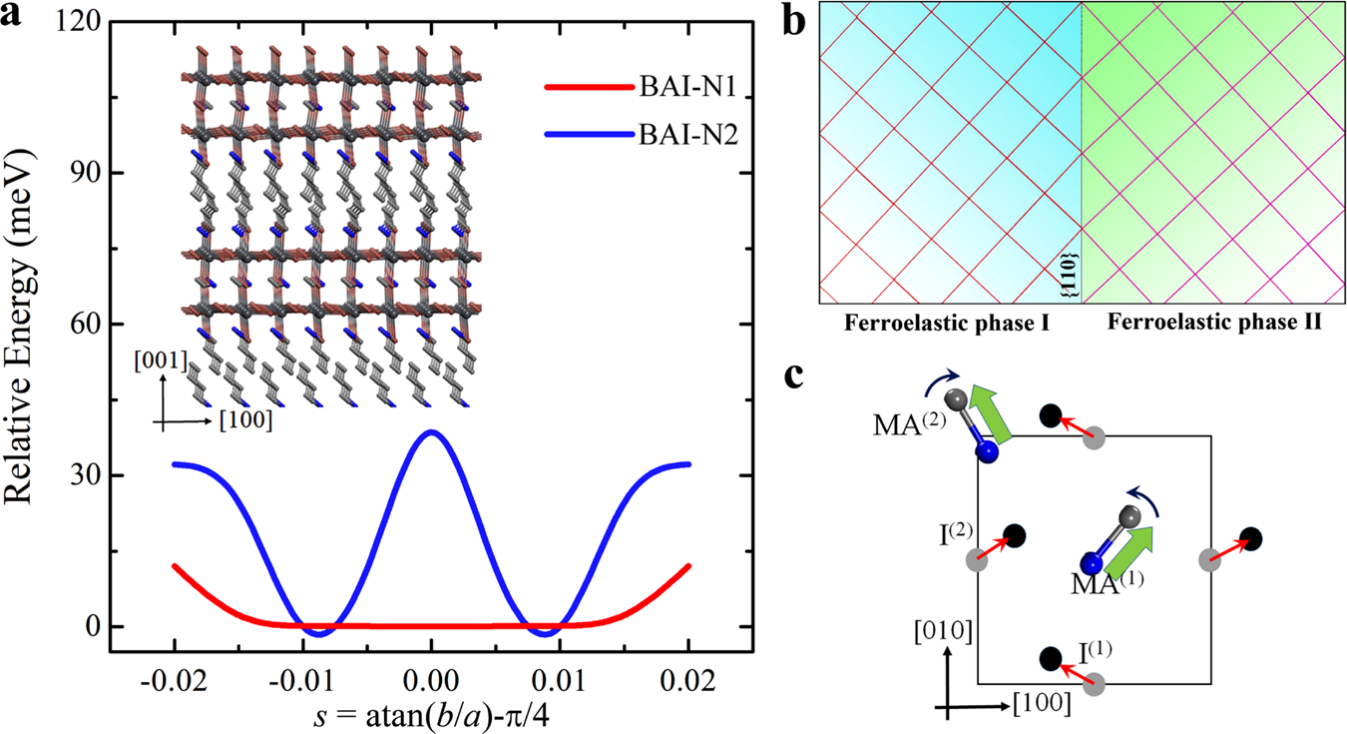
DFT results of ferroelasticity in layered perovskites. **a** Relative energy per simulation supercell with respect to ferroelastic strain *s* for BAI-N1 (red curve) and BAI-N2 (blue curve) structures. The inset shows the relaxed atomic geometric structure (hydrogen atoms are removed) of BAI-N2. **b** Ferroelastic domain between two phases. **c** MA rotations induced ferroelasticity. The solid gray and black circles represent I atoms before and after distortions, and the green arrows show the polarization of MA molecules.

In order to understand the ferroelasticity mechanism and identify the twin structures of these layered perovskites, we perform first-principles density functional theory (DFT) calculations, as implemented in the Vienna ab initio simulation package (VASP) code. We used various geometric structures as initial configurations and performed full geometric relaxations for both the Bravais lattice vectors ({**H**_i_}, *i* = 1, 2, 3) and the internal coordinates ({**s**_*j*_}, *j* = 1, 2, …, *N*, where *N* is the total number of atoms in one simulation supercell)^34^. Due to the intense computational cost in DFT calculations, here we focus on the BAI-N1 and BAI-N2 crystals. Each simulation supercell contains two I-Pb-I layers separated by one butylammonium (BA) molecular layer. Thus, the chemical formula in one supercell are H_96_C_32_N_8_Pb_4_I_16_ (BAI-N1) and H_120_C_36_N_12_Pb_8_I_28_ (BAI-N2). After careful relaxations and comparison with previous theoretical and experimental geometries^30^, we found a tetragonal Bravais lattice (*a* = *b* = 8.55 Å, *c* = 27.53 Å) in the BAI-N1 system to be energetically favorable. We calculated the total energy curve as a function of ferroelastic strain *s* (= atan(*b*/*a*) − π/4), which shows a relatively flat basin with *s* in the range of (−0.01, +0.01) (Fig. 3a). This suggests that at finite temperature, the tetragonal structure can only be observed and a phase transition to orthorhombic lattice would not occur. In contrast, geometric relaxation shows that the ground state of BAI-N2 layered perovskite has an orthorhombic Bravais lattice, with lattice constants *a* = 8.51 Å, *b* = 8.68 Å, and *c* = 40.90 Å (atomic structure shown inset of Fig. 3a). The parental phase is tetragonal with its (Pb-I skeleton) space group of I4/mmm, while the space group is lowered to *Pnma* at lower temperature. According to Aizu’s classification^35^, the spontaneous strain could enable a transition denoted as 4/*mmm*F*mmm*, where the point groups before and after “F” are the parental and ferroic phases, respectively. It corresponds to a pure and full ferroelastic phase transition, without ferroelectricity. As seen in Supplementary Figure 6, there are two ferroelastic phases generated from the parental 4/*mmm* structure, which can be denoted as X and Y. To preserve crystal integrity, different domains have to share their *c* axes, and the domain boundary is parallel to the *c* axis. To be specific, the difference in the lattice spacing between {1 0 0} and {0 1 0} planes (*d*_{010}_ *< d*_{100}_) reproduces the experimentally observed ferroelastic domains. The mirror plane of the twinning domain should be parallel to {1 1 0} (Fig. 3b). Thus, the change of ferroelastic strain can be evaluated to be *s*_0_ = 0.01 (=0.57°). This indicates that adjacent ferroelastic domain walls always form an angle of 90° – 2*s*_0_ = 88.9°, consistent with the experimental observations of domains intersection angle (~89°) on BAI-N2 crystals top surface (Fig. 2c). We also calculate total energy variation as a function of ferroelastic strain, where we see that the paraelastic (tetragonal lattice, *s* = 0) structure serves as a saddle point on the energy curve. This paraelastic system separates two equivalent ferroelastic structures ±*s*_0_ with an energy barrier of 40 meV/supercell (i.e., 2.1 J/cm^3^).

Next, we explore the ferroelasticity mechanism in the BAI-N2 layered organic perovskites. Note that traditional ferroelasticity is usually attributed to the displacive movement of a group of atoms, here we illustrate another ferroelasticity mechanism owing to the rotation of MA molecules. Because of the aspherical shape of the MA molecules, the Pb_*n*_I_3*n*+1_ (*n* = 2) framework is distorted, which has been well reported for 3D MAPI crystals both theoretically and experimentally^36–38^. We focus on the iodine atoms on the same *X-Y* plane with MA molecules. Two inequivalent iodine atoms are considered (Fig. 3c), namely, I^(1)^ and I^(2)^. Before distortion, their coordinates can be written as I^(1)^ = (0.5 × *a*, 0, *z*^(1)^) and I^(2)^ = (0, 0.5 × *b*, *z*^(2)^) (shown as solid light gray circles). Naively speaking, the MA molecules could lie along the high symmetric <110> direction. The Pb_*n*_I_3*n*+1_ framework distortion changes their coordinates in the *X-Y*-plane, which can be written as I^(1)^ = (0.5 × *a* – *δ*^(1)^ × cos*θ*^(1)^, *δ*^(1)^ × sin*θ*^(1)^, *z*^(1)^) and I^(2)^ = (*δ*^(2)^ × cos*θ*^(2)^, 0.5 × *b* + *δ*^(1)^ × sin*θ*^(2)^, *z*^(2)^), where *δ* and *θ* are displacement magnitude and its angle with the *x*-axis. From our DFT results, the average values of *δ*s and *θ*s are about 0.4 Å and 15°, respectively. Such displacive movements change the position of MA molecules as well as their directions due to strong steric interactions. Note in Fig. 3c that the MA molecules with NH_3_ head and CH_3_ tail rotate towards the *Y*-axis, along with shuffling of iodine with respect to the Bravais lattice. Our DFT calculations show an average angle between the MA and the *y*-axis to be 33°. Note that similar MA molecule rotations at low temperatures have also been proposed theoretically in 3D MAPbI_3_ crystals^37,38^, agreeing with our DFT calculations for layered perovskites. This rotation of the aspherical MA molecules breaks the strain equivalence between the lattice constants *a* and *b*, resulting in ferroelasticity in the *n* ≥ 2 layered perovskites. On the other hand, the absence of MA molecules in the BAI-N1 structures retains its tetragonal (*a* = *b*) Bravais lattice feature, consistent with our experimental results.

### Impact of ferroelastic domains on carrier recombination

**Fig. 4:**
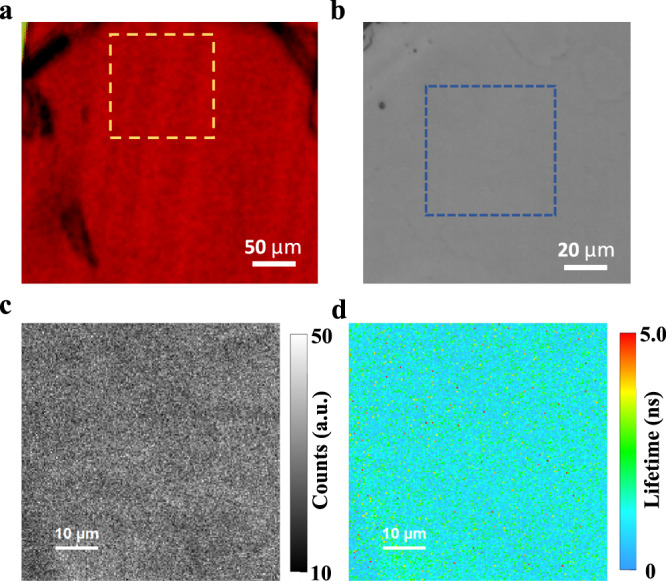
PL intensity and recombination lifetime mapping for ferroelastic domains. **a** is polarized optical microscope image with resolved domain patterns, the golden square corresponds to the non-polarized optical microscope image in (**b**); the blue square in (**b**) is the mapping area; **c** and **d** are the corresponding PL intensity and PL recombination lifetime mapping results, respectively.

After the confirmation of ferroelasticity in layered perovskites with *n *> 1, we studied the impact of ferroelastic domains on carrier recombination kinetics in layered perovskites with confocal PL intensity and PL recombination lifetime mapping. A 405 nm pulsed laser with a repetition rate of 20 MHz was focused onto the sample surface by a 50 × objective lens (NA = 0.5). The excitation spot size could reach a near-diffraction limit of approximately 500 nm and the excitation carrier density is ~6.3 × 10^12^ cm^−2^. Photoluminescence was then collected through the same lens to an avalanche photodiode for time-correlated single-photon counting. By scanning the excitation lens along the *X*- and *Y*- axis with a piezo-driven motor, the mapping results for PL intensity and recombination lifetime could be acquired with the PicoQuant Fluo Time 300 system. A BAI-N2 single crystal sample with ferroelastic domains identified by polarized optical microscopy was selected for use in these studies, as shown in Fig. 4a. The location with clear ferroelastic domain patterns is marked with a golden square in Fig. 4a, which corresponds to the non-polarized optical microscope image in Fig. 4b. As shown in Fig. 4c, the PL intensity exhibits a uniform distribution over the mapping area of the blue square region in Fig. 4b. Considering the existence of ferroelastic domains in this area, it is evident that the ferroelastic twin structures in layered perovskite do not induce non-radiative recombination or reduce the PL quantum yield significantly. The PL recombination lifetime mapping is acquired at the same area as shown in Fig. 4d, again showing no obvious change at the ferroelastic domains. As neither PL intensity nor PL lifetime exhibits a significant change at ferroelastic domains, it suggests that the ferroelastic domain walls (twin boundaries) do not behave as non-radiative recombination centers, likely due to the high degree of structural coherency of the twin boundary. Also, while the MA molecular orientation sustains an abrupt jump from ~33° to ~57° across the twin boundary, because the near-band gap electronic structure is dominated by the inorganic Pb *s*-*p* and I *p* electronic orbitals^39^ while the organics mainly serve as structural support, this abrupt jump clearly does not disturb the carrier wavefunctions that much, that need to cross to the other side of the twin boundary^40^.

## Discussion

In conclusion, we reported solid evidences of ferroelasticity in layered perovskites with in situ strain-driven domain motion. The ferroelasticity in layered perovskites was found to be layer number dependent. Fundamental investigation of the ferroelasticity in layered perovskites by DFT calculations suggested that the rotation of aspherical methylammonium cations breaks the strain equivalence and causes the ferroelasticity. The layer number dependence of ferroelasticity for *n* = 1 and *n* ≥ 2 layered perovskites then results from the compositional difference or the presence of methylammonium cations. Finally, the ferroelastic domains in layered perovskites were determined to not behave as non-radiative recombination pathways and have a negligible effect on carrier recombination kinetics. Our findings provide scientific insights in understanding strain-dependent material properties in 2D perovskites and lead to new potential flexible electronics controlled by strain engineering.

## Methods

### Materials

The materials were used as follows: lead iodide (PbI_2_) (>99.999%, Alfa Aesar), n-Butylammonium iodide (BAI) (great cell solar), hydriodic acid (HI) (48% w/w aq. soln. Alfa Aesar), methylammonium iodide (MAI) (great cell solar).

### Growth of thin layered perovskites single crystals

Solutions for BAI-N1, BAI-N2, and BAI-N3 crystal growth were prepared by dissolving BAI, MAI, and PbI_2_, with respective molar ratios of 2:0:1, 2:1:2, and 2:2:3, in HI at 130 °C. After inserting 150 μL precursor between two glass substrates (2 × 3 inches) at 130 °C, the growth temperature was gradually decreased to ~20 °C at a rate of 10 °C per h to produce thin layered perovskite crystals.

### Density functional theory

Our studies are based on density functional theory (DFT) and the projector augment wave (PAW) method as implemented in the Vienna ab initio Simulation Package (VASP). The valance electron wave functions are expanded using planewave basis sets with a kinetic energy cutoff of 400 eV. The electronic exchange-correlation functional in the form of PBE is used. The integration in the reciprocal space is replaced by the summation over ***k***-points sampled by the Γ-point-centered Monkhorst-Pack scheme with a grid density of 2π × 0.02 Å^−1^. A conjugate gradient algorithm is used to fully optimize the lattice constants and the internal atomic geometries. Convergence criteria for total energy and Hellmann–Feynman force components are set at 10^−5^ eV and 10^−2^ eV/Å, respectively.

## Supplementary information

Supplementary Information

## Data Availability

The data that support this study are available from the corresponding author upon reasonable request.
