# Supplementary material for: Layer number dependent ferroelasticity in 2D Ruddlesden–Popper organic-inorganic hybrid perovskites
Source: Nat Commun. 2021 Feb 26;12:1332. doi: 10.1038/s41467-021-21493-w (PMC7910601; doi:10.1038/s41467-021-21493-w)
Supplement: Supplementary file 1 — Supplementary Information [file 41467_2021_21493_MOESM1_ESM.pdf]

# Supplementary Information for

## Layer Number Dependent Ferroelasticity in 2D Ruddlesden-Popper Organic-inorganic Hybrid Perovskites

Xun Xiao[1], Jian Zhou[2], Kepeng Song[3], Jingjing Zhao[1], Yu Zhou[1], Peter Neil Rudd[1], Yu Han[3], Ju Li[2,*] and Jinsong Huang[1,*]

[1] *Department of Applied Physical Sciences, University of North Carolina, Chapel Hill, NC 27599, USA.*

[2] *Department of Nuclear Science and Engineering and Department of Materials Science and Engineering, Massachusetts Institute of Technology, Cambridge, MA 02139, USA.*

[3] *Advanced Membranes and Porous Materials (AMPM) Center, Physical Sciences and Engineering Division, King Abdullah University of Science and Technology (KAUST), Thuwal, Saudi Arabia*

[*] Correspondence to J.H. (email: jhuang@unc.edu) or to J.L. (email: liju@mit.edu)

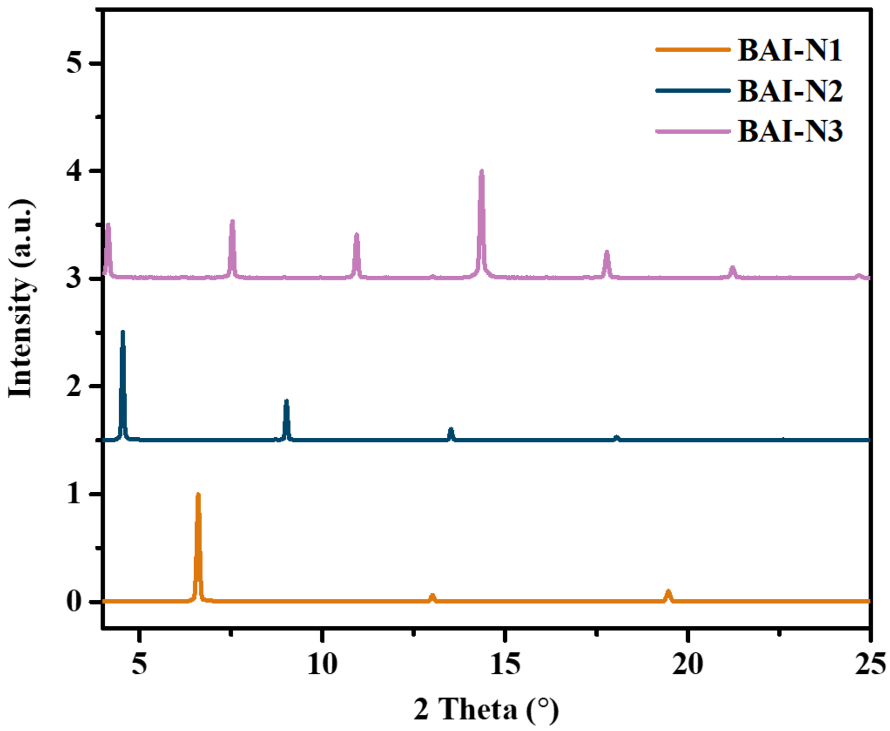


**Supplementary Figure 1:** XRD patterns for BAI-N1, BAI-N2 and BAI-N3 single crystals, respectively.

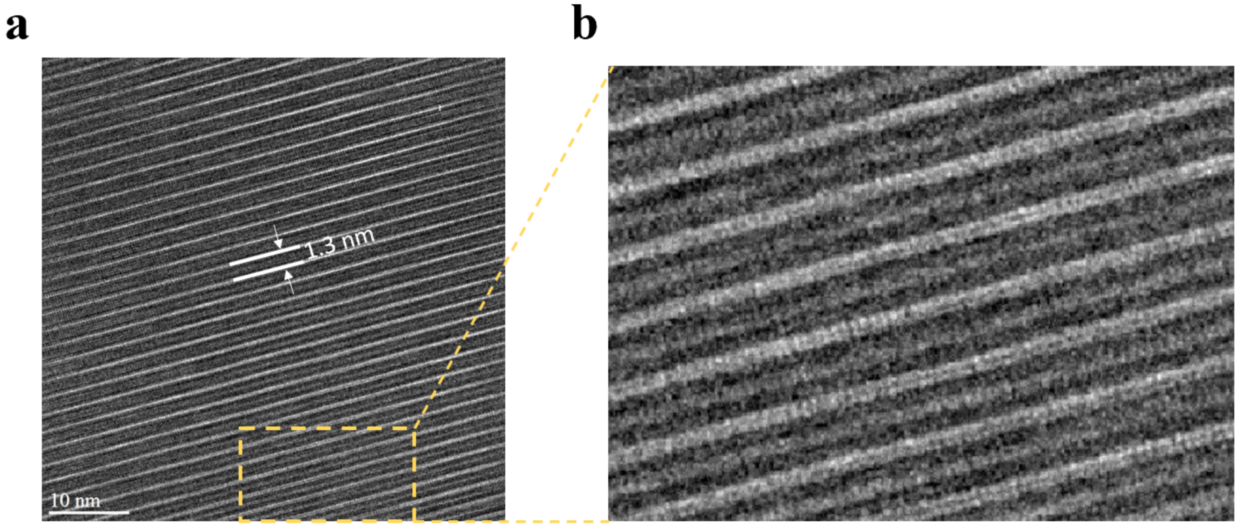


**Supplementary Figure 2: (a)** Cross section TEM for BAI-N2, **(b)** is the zoom-in image corresponding to golden rectangular mark in **(a)**.

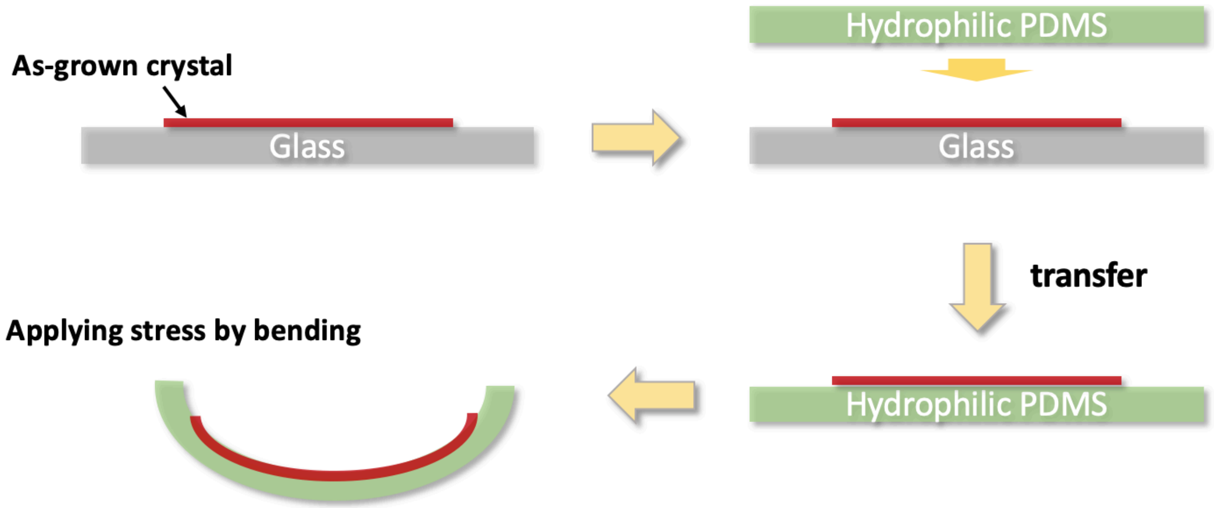


**Supplementary Figure 3:** Scheme of applying external stress on layered perovskite crystals

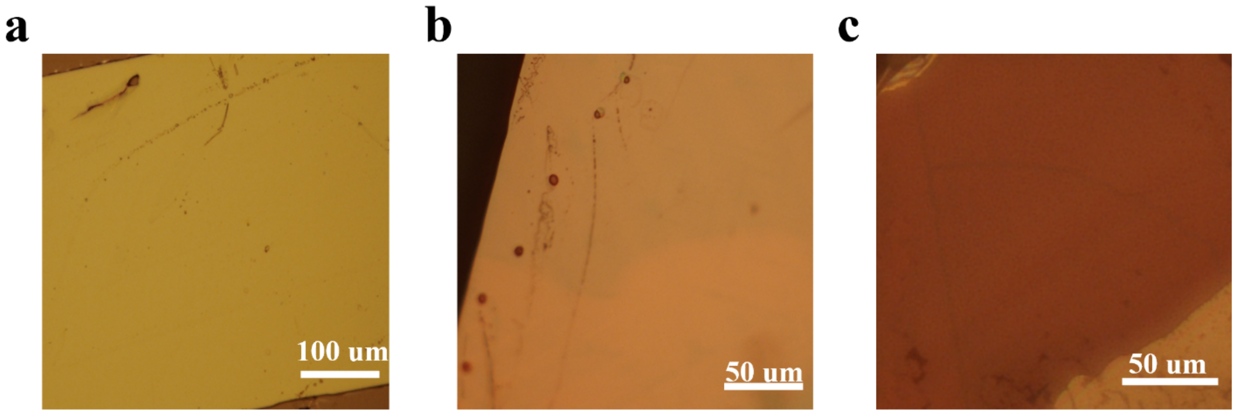


**Supplementary Figure 4: (a)**, **(b)** and **(c)** are non-polarized optical microscope images for BAI-N1, BAI-N2 and BAI-N3 single crystals, respectively.

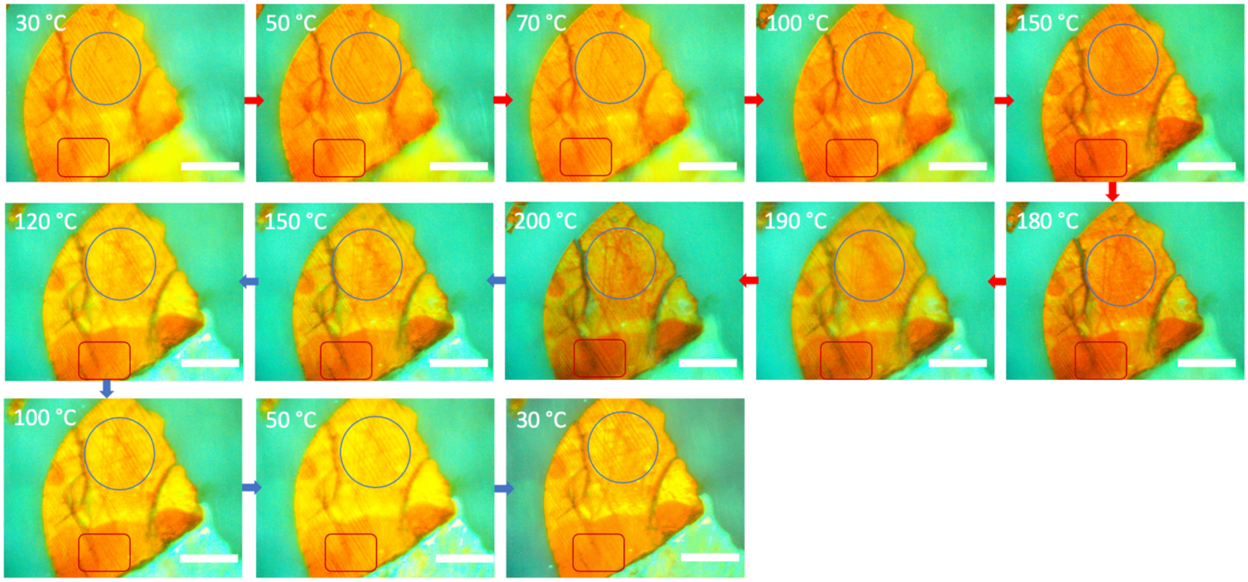


**Supplementary Figure 5:** Temperature-dependent microscopy of BAI-N2. Scale bar of 200 µm.

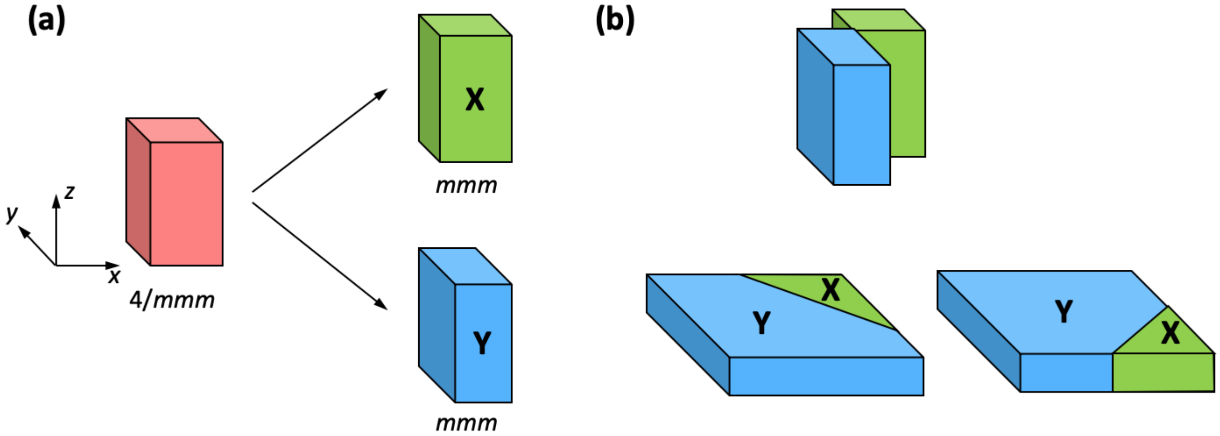


**Supplementary Figure 6: (a)** Space group change of BAI-N2 from tetragonal-to-orthorhombic transition; **(b)** permissible domains configuration

**Supplementary Note 1: DFT calculation models for BAI-N1 and BAI-N2.**

**BAI-N1 (VASP form)**

```
BAI-N1
1.0000000000
    8.55045    0.00000    0.000-0
    0.00000    8.55045    0.00000
    0.00000    0.00000   27.53405
  H    C    N    Pb   I
  96   32    8    4   16
  Direct
  0.264512    0.434668    0.722538
  0.448592    0.219584    0.732024
  0.324343    0.318234    0.808609
  0.340386    0.959671    0.806062
  0.206742    0.058346    0.881770
  0.127183    0.291085    0.504313
  0.337771    0.088998    0.511514
  0.341462    0.293102    0.419305
  0.571102    0.118750    0.418764
  0.409909    0.119418    0.340055
  0.419348    0.785804    0.234368
  0.228105    0.569407    0.230342
  0.331166    0.650854    0.311553
  0.976041    0.598218    0.316671
  0.111149    0.652115    0.398157
  0.048159    0.767957    0.025720
  0.243944    0.559132    0.002678
  0.160509    0.814605    0.928055
  0.360865    0.630670    0.895265
  0.140022    0.714567    0.839713
  0.609833    0.695036    0.719338
  0.786989    0.919224    0.724617
  0.659479    0.808683    0.800467
  0.997433    0.920135    0.816562
  0.866364    0.812146    0.892932
  0.761190    0.572377    0.506677
  0.952090    0.780775    0.505935
  0.700299    0.829664    0.427189
  0.912974    0.039499    0.433045
  0.787302    0.979469    0.352820
  0.769325    0.053132    0.237126
  0.566141    0.246391    0.238012
  0.691376    0.185798    0.320194
  0.580734    0.520869    0.301025
```

| | | |
|---|---|---|
| 0.734967 | 0.517728 | 0.378039 |
| 0.692234 | 0.081292 | 0.019149 |
| 0.870840 | 0.264780 | 0.986111 |
| 0.541370 | 0.339139 | 0.946510 |
| 0.752064 | 0.523875 | 0.917452 |
| 0.532423 | 0.450864 | 0.860344 |
| 0.762813 | 0.569270 | 0.723503 |
| 0.945431 | 0.786467 | 0.732416 |
| 0.823515 | 0.685290 | 0.809048 |
| 0.836008 | 0.044082 | 0.807266 |
| 0.707277 | 0.940451 | 0.883264 |
| 0.610521 | 0.701575 | 0.505419 |
| 0.798131 | 0.920666 | 0.508319 |
| 0.849973 | 0.687211 | 0.424635 |
| 0.053816 | 0.892338 | 0.423570 |
| 0.924781 | 0.830351 | 0.341787 |
| 0.912681 | 0.190051 | 0.236724 |
| 0.707522 | 0.396775 | 0.229671 |
| 0.822490 | 0.342957 | 0.310869 |
| 0.465221 | 0.362941 | 0.321003 |
| 0.623280 | 0.359512 | 0.400348 |
| 0.549459 | 0.214127 | 0.025669 |
| 0.749394 | 0.422825 | 0.004542 |
| 0.656385 | 0.178792 | 0.927400 |
| 0.862369 | 0.362126 | 0.896686 |
| 0.642470 | 0.287148 | 0.839823 |
| 0.112177 | 0.308058 | 0.718915 |
| 0.292039 | 0.084904 | 0.723852 |
| 0.161505 | 0.193733 | 0.799464 |
| 0.499857 | 0.085197 | 0.816528 |
| 0.363233 | 0.189010 | 0.892620 |
| 0.267538 | 0.430494 | 0.502144 |
| 0.474526 | 0.242022 | 0.500846 |
| 0.214193 | 0.134403 | 0.430924 |
| 0.457477 | 0.960448 | 0.439571 |
| 0.300242 | 0.960844 | 0.363145 |
| 0.270406 | 0.917011 | 0.234129 |
| 0.076512 | 0.711558 | 0.234524 |
| 0.186074 | 0.797631 | 0.315859 |
| 0.112326 | 0.447982 | 0.305744 |
| 0.244021 | 0.499944 | 0.385478 |
| 0.191871 | 0.900202 | 0.020173 |
| 0.369817 | 0.716307 | 0.986520 |
| 0.040077 | 0.655745 | 0.944990 |
| 0.248481 | 0.468673 | 0.914666 |
| 0.029530 | 0.549085 | 0.858577 |

| | | |
|---|---|---|
| 0.251155 | 0.309781 | 0.675787 |
| 0.395661 | 0.984524 | 0.895879 |
| 0.251564 | 0.314222 | 0.550735 |
| 0.501798 | 0.935460 | 0.349846 |
| 0.297626 | 0.796854 | 0.186889 |
| 0.042839 | 0.456095 | 0.394353 |
| 0.209424 | 0.752901 | 0.060562 |
| 0.205596 | 0.524893 | 0.825273 |
| 0.750020 | 0.692875 | 0.676546 |
| 0.895676 | 0.017008 | 0.896717 |
| 0.731159 | 0.695941 | 0.553240 |
| 0.987761 | 0.028303 | 0.344854 |
| 0.791335 | 0.171447 | 0.189405 |
| 0.532966 | 0.544150 | 0.390512 |
| 0.711008 | 0.225480 | 0.060526 |
| 0.709320 | 0.478124 | 0.827698 |
| 0.324028 | 0.198550 | 0.739693 |
| 0.289042 | 0.203687 | 0.793805 |
| 0.372390 | 0.073727 | 0.821801 |
| 0.332849 | 0.076387 | 0.875783 |
| 0.356264 | 0.199937 | 0.492130 |
| 0.333240 | 0.179125 | 0.437811 |
| 0.453709 | 0.065812 | 0.416598 |
| 0.415331 | 0.018142 | 0.364624 |
| 0.196060 | 0.682835 | 0.246305 |
| 0.209830 | 0.679925 | 0.301283 |
| 0.097512 | 0.561544 | 0.324018 |
| 0.124360 | 0.541357 | 0.378341 |
| 0.246567 | 0.679631 | 0.988659 |
| 0.163140 | 0.691674 | 0.940190 |
| 0.239834 | 0.590128 | 0.901295 |
| 0.148921 | 0.594513 | 0.853716 |
| 0.820736 | 0.805908 | 0.740338 |
| 0.786935 | 0.799789 | 0.794509 |
| 0.870105 | 0.929900 | 0.822488 |
| 0.833354 | 0.924813 | 0.876625 |
| 0.832735 | 0.806600 | 0.493347 |
| 0.821816 | 0.805032 | 0.438298 |
| 0.931826 | 0.925373 | 0.415446 |
| 0.907565 | 0.941817 | 0.360875 |
| 0.684595 | 0.284834 | 0.248002 |
| 0.703294 | 0.301025 | 0.302661 |
| 0.582922 | 0.414897 | 0.323740 |
| 0.619576 | 0.461218 | 0.375943 |
| 0.748576 | 0.303990 | 0.989194 |
| 0.663118 | 0.300030 | 0.940919 |

| | | |
|---|---|---|
| 0.741831 | 0.403878 | 0.902958 |
| 0.651690 | 0.405414 | 0.855206 |
| 0.232763 | 0.320164 | 0.712924 |
| 0.243822 | 0.315376 | 0.513121 |
| 0.302030 | 0.802608 | 0.224500 |
| 0.169601 | 0.780869 | 0.025884 |
| 0.730615 | 0.683335 | 0.713644 |
| 0.727652 | 0.686882 | 0.515659 |
| 0.796039 | 0.168311 | 0.227043 |
| 0.670836 | 0.200330 | 0.025663 |
| 0.208855 | 0.238425 | 0.125609 |
| 0.709777 | 0.737072 | 0.124658 |
| 0.312015 | 0.751780 | 0.612859 |
| 0.815755 | 0.253838 | 0.612876 |
| 0.524211 | 0.053345 | 0.134787 |
| 0.023966 | 0.921793 | 0.134976 |
| 0.878726 | 0.406471 | 0.119273 |
| 0.379588 | 0.567637 | 0.117064 |
| 0.237507 | 0.192740 | 0.009316 |
| 0.736560 | 0.786833 | 0.008461 |
| 0.713027 | 0.761924 | 0.243246 |
| 0.206622 | 0.204089 | 0.244574 |
| 0.987130 | 0.580952 | 0.615417 |
| 0.135188 | 0.074088 | 0.613030 |
| 0.631025 | 0.935218 | 0.615055 |
| 0.490350 | 0.428195 | 0.613812 |
| 0.829992 | 0.291412 | 0.495869 |
| 0.314673 | 0.717190 | 0.495699 |
| 0.318421 | 0.720981 | 0.730304 |
| 0.819519 | 0.281865 | 0.730435 |

**BAI-N2 (VASP form)**

```
BAI-N2
  1.00000000000000
    8.51265    0.00000    0.00000
    0.00000    8.68462    0.00000
    0.00000    0.00000   40.90000
  H    C    N    Pb   I
  120    36    12     8    28
Direct
   0.25061       0.43368           0.48401
   0.43541       0.22094           0.49147
   0.29916       0.32052           0.54169
   0.31859       0.96732           0.54068
   0.17774       0.06523           0.59089
```

| | | |
|---|---|---|
| 0.10986 | 0.28835 | 0.18262 |
| 0.31148 | 0.08332 | 0.18376 |
| 0.32191 | 0.29575 | 0.12333 |
| 0.54033 | 0.10618 | 0.12198 |
| 0.38802 | 0.14190 | 0.06850 |
| 0.40375 | 0.78299 | 0.98786 |
| 0.21453 | 0.56789 | 0.98748 |
| 0.32640 | 0.65669 | 0.04107 |
| 0.97221 | 0.59298 | 0.04668 |
| 0.10784 | 0.66079 | 0.10071 |
| 0.03217 | 0.77076 | 0.69521 |
| 0.21450 | 0.55813 | 0.67907 |
| 0.15920 | 0.81925 | 0.62924 |
| 0.35121 | 0.62507 | 0.60830 |
| 0.14571 | 0.72675 | 0.56886 |
| 0.59460 | 0.69617 | 0.47962 |
| 0.76768 | 0.91817 | 0.48510 |
| 0.63660 | 0.80459 | 0.53531 |
| 0.97480 | 0.91389 | 0.54790 |
| 0.83914 | 0.80488 | 0.59854 |
| 0.74721 | 0.57046 | 0.18127 |
| 0.93732 | 0.77881 | 0.17941 |
| 0.67519 | 0.81817 | 0.12801 |
| 0.87981 | 0.03202 | 0.12949 |
| 0.75667 | 0.95835 | 0.07570 |
| 0.76259 | 0.05352 | 0.99215 |
| 0.56191 | 0.24894 | 0.99369 |
| 0.68458 | 0.17772 | 0.04817 |
| 0.60210 | 0.51895 | 0.03870 |
| 0.74462 | 0.48284 | 0.09187 |
| 0.68648 | 0.08967 | 0.68872 |
| 0.86077 | 0.27648 | 0.66614 |
| 0.52798 | 0.32690 | 0.63810 |
| 0.72910 | 0.52204 | 0.61910 |
| 0.51593 | 0.43673 | 0.58006 |
| 0.74806 | 0.57342 | 0.48348 |
| 0.92857 | 0.78983 | 0.49067 |
| 0.80137 | 0.68314 | 0.54122 |
| 0.81333 | 0.03667 | 0.54134 |
| 0.68010 | 0.93252 | 0.59191 |
| 0.59573 | 0.69696 | 0.18219 |
| 0.78047 | 0.91388 | 0.18184 |
| 0.82961 | 0.68214 | 0.12559 |
| 0.02769 | 0.89237 | 0.12402 |
| 0.90257 | 0.81703 | 0.06950 |
| 0.90795 | 0.18680 | 0.99035 |

| | | |
|---|---|---|
| 0.70812 | 0.39353 | 0.98848 |
| 0.82660 | 0.32436 | 0.04262 |
| 0.46971 | 0.37026 | 0.04868 |
| 0.61531 | 0.33240 | 0.10292 |
| 0.54010 | 0.21663 | 0.69296 |
| 0.73024 | 0.42699 | 0.67731 |
| 0.65559 | 0.17678 | 0.62607 |
| 0.85065 | 0.37002 | 0.60551 |
| 0.63745 | 0.28282 | 0.56653 |
| 0.10068 | 0.30820 | 0.47901 |
| 0.27840 | 0.08936 | 0.48458 |
| 0.13825 | 0.19600 | 0.53438 |
| 0.47696 | 0.09235 | 0.54845 |
| 0.33354 | 0.19521 | 0.59874 |
| 0.25249 | 0.42270 | 0.17916 |
| 0.45560 | 0.22838 | 0.17704 |
| 0.18376 | 0.14694 | 0.13027 |
| 0.41097 | 0.95601 | 0.13272 |
| 0.26187 | 0.98991 | 0.08026 |
| 0.25406 | 0.91076 | 0.98844 |
| 0.06086 | 0.70584 | 0.99077 |
| 0.17675 | 0.79718 | 0.04452 |
| 0.11483 | 0.44938 | 0.03995 |
| 0.24737 | 0.51457 | 0.09313 |
| 0.18029 | 0.89624 | 0.69104 |
| 0.35394 | 0.70420 | 0.66970 |
| 0.02461 | 0.67112 | 0.63892 |
| 0.22341 | 0.47370 | 0.61949 |
| 0.01979 | 0.57164 | 0.57973 |
| 0.24922 | 0.31444 | 0.45184 |
| 0.36694 | 0.99423 | 0.60133 |
| 0.24447 | 0.30987 | 0.21228 |
| 0.46315 | 0.95189 | 0.07232 |
| 0.27542 | 0.78996 | 0.95670 |
| 0.04738 | 0.46500 | 0.09970 |
| 0.19449 | 0.75202 | 0.71852 |
| 0.19993 | 0.53586 | 0.55923 |
| 0.73993 | 0.69547 | 0.45174 |
| 0.86918 | 0.00624 | 0.60163 |
| 0.72490 | 0.69088 | 0.21310 |
| 0.95554 | 0.01492 | 0.07026 |
| 0.77873 | 0.16991 | 0.95969 |
| 0.54293 | 0.52334 | 0.09878 |
| 0.70353 | 0.23463 | 0.71604 |
| 0.69311 | 0.47501 | 0.55865 |
| 0.70655 | 0.37135 | 0.81672 |

| | | |
|---|---|---|
| 0.74596 | 0.09481 | 0.82126 |
| 0.28151 | 0.88094 | 0.86468 |
| 0.20934 | 0.61551 | 0.85510 |
| 0.26719 | 0.91521 | 0.82147 |
| 0.20271 | 0.64363 | 0.81469 |
| 0.70414 | 0.38102 | 0.86059 |
| 0.74346 | 0.10527 | 0.86209 |
| 0.55086 | 0.28014 | 0.83923 |
| 0.41651 | 0.78919 | 0.83721 |
| 0.06888 | 0.72864 | 0.83898 |
| 0.89093 | 0.18992 | 0.84087 |
| 0.62254 | 0.70344 | 0.31064 |
| 0.89866 | 0.77194 | 0.31145 |
| 0.12209 | 0.30594 | 0.35335 |
| 0.39761 | 0.23555 | 0.35313 |
| 0.12744 | 0.30757 | 0.30943 |
| 0.40227 | 0.23638 | 0.31227 |
| 0.62570 | 0.70257 | 0.35456 |
| 0.90093 | 0.77229 | 0.35230 |
| 0.73942 | 0.56405 | 0.33210 |
| 0.24090 | 0.44489 | 0.33223 |
| 0.29466 | 0.10266 | 0.33205 |
| 0.79519 | 0.90602 | 0.33209 |
| 0.30920 | 0.20105 | 0.49551 |
| 0.26684 | 0.20695 | 0.53162 |
| 0.34862 | 0.08028 | 0.55126 |
| 0.30465 | 0.08385 | 0.58741 |
| 0.33421 | 0.19374 | 0.17148 |
| 0.30652 | 0.18186 | 0.13484 |
| 0.41839 | 0.06529 | 0.11911 |
| 0.38155 | 0.03594 | 0.08316 |
| 0.18256 | 0.68016 | 0.99799 |
| 0.20259 | 0.68118 | 0.03493 |
| 0.09598 | 0.56258 | 0.05148 |
| 0.12556 | 0.55012 | 0.08817 |
| 0.22765 | 0.67696 | 0.67014 |
| 0.15106 | 0.69764 | 0.63689 |
| 0.22601 | 0.59431 | 0.61095 |
| 0.14353 | 0.60759 | 0.57796 |
| 0.80250 | 0.80641 | 0.49547 |
| 0.76500 | 0.79670 | 0.53177 |
| 0.84663 | 0.92348 | 0.55140 |
| 0.80690 | 0.91652 | 0.58774 |
| 0.81567 | 0.80170 | 0.17175 |
| 0.79903 | 0.79776 | 0.13476 |
| 0.90371 | 0.91870 | 0.11843 |

| | | |
|---|---|---|
| 0.87893 | 0.92775 | 0.08156 |
| 0.68248 | 0.28244 | 0.00023 |
| 0.70407 | 0.29184 | 0.03706 |
| 0.59175 | 0.40964 | 0.05221 |
| 0.62473 | 0.43837 | 0.08834 |
| 0.73596 | 0.30890 | 0.66765 |
| 0.65301 | 0.29680 | 0.63488 |
| 0.72688 | 0.40339 | 0.60940 |
| 0.63866 | 0.39952 | 0.57696 |
| 0.67586 | 0.31024 | 0.83924 |
| 0.29586 | 0.83052 | 0.84035 |
| 0.69436 | 0.68222 | 0.33236 |
| 0.19512 | 0.32699 | 0.33180 |
| 0.22239 | 0.32138 | 0.47661 |
| 0.22881 | 0.31017 | 0.18707 |
| 0.28490 | 0.79793 | 0.98196 |
| 0.15435 | 0.77993 | 0.69515 |
| 0.71630 | 0.68555 | 0.47659 |
| 0.71482 | 0.68287 | 0.18784 |
| 0.78958 | 0.16646 | 0.98497 |
| 0.66234 | 0.20652 | 0.69277 |
| 0.18709 | 0.69732 | 0.83717 |
| 0.77026 | 0.16626 | 0.84095 |
| 0.32982 | 0.21800 | 0.33234 |
| 0.82975 | 0.79046 | 0.33204 |
| 0.20285 | 0.24814 | 0.75810 |
| 0.21718 | 0.24416 | 0.91849 |
| 0.70103 | 0.75173 | 0.75911 |
| 0.71608 | 0.74082 | 0.91857 |
| 0.27585 | 0.75037 | 0.25231 |
| 0.77942 | 0.24863 | 0.25210 |
| 0.28086 | 0.75716 | 0.41099 |
| 0.78139 | 0.25338 | 0.41089 |
| 0.52539 | 0.06995 | 0.77015 |
| 0.01530 | 0.94163 | 0.76785 |
| 0.88104 | 0.43062 | 0.75023 |
| 0.36797 | 0.58244 | 0.75832 |
| 0.23172 | 0.18849 | 0.68102 |
| 0.71993 | 0.79791 | 0.68156 |
| 0.90051 | 0.42811 | 0.92319 |
| 0.39010 | 0.56764 | 0.91265 |
| 0.54618 | 0.06755 | 0.91338 |
| 0.03457 | 0.92810 | 0.91619 |
| 0.75987 | 0.73720 | 0.83952 |
| 0.21028 | 0.19574 | 0.99673 |
| 0.69875 | 0.77383 | 0.99735 |

| | | |
|---|---|---|
| 0.17682 | 0.27712 | 0.83844 |
| 0.95149 | 0.57377 | 0.25985 |
| 0.09992 | 0.06965 | 0.25140 |
| 0.59660 | 0.93289 | 0.25129 |
| 0.45310 | 0.42768 | 0.25945 |
| 0.80892 | 0.29110 | 0.17413 |
| 0.29530 | 0.70603 | 0.17412 |
| 0.60277 | 0.93610 | 0.41261 |
| 0.45325 | 0.42915 | 0.40512 |
| 0.95465 | 0.57923 | 0.40527 |
| 0.10276 | 0.07381 | 0.41233 |
| 0.26129 | 0.80401 | 0.33175 |
| 0.80192 | 0.29002 | 0.48928 |
| 0.29640 | 0.71826 | 0.48940 |
| 0.76135 | 0.20382 | 0.33164 |
